# Evaluation of current prediction models for Lynch syndrome: updating the PREMM5 model to identify PMS2 mutation carriers

**DOI:** 10.1007/s10689-017-0039-1

**Published:** 2017-09-20

**Authors:** A. Goverde, M. C. W. Spaander, D. Nieboer, A. M. W. van den Ouweland, W. N. M. Dinjens, H. J. Dubbink, C. J. Tops, S. W. ten Broeke, M. J. Bruno, R. M. W. Hofstra, E. W. Steyerberg, A. Wagner

**Affiliations:** 1 Department of Clinical Genetics, Erasmus MC, University Medical Center, Rotterdam, The Netherlands; 2 Department of Gastroenterology and Hepatology, Erasmus MC, University Medical Center, Rotterdam, The Netherlands; 3 Department of Public Health, Erasmus MC, University Medical Center, Rotterdam, The Netherlands; 4 Department of Pathology, Erasmus MC, University Medical Center, Rotterdam, The Netherlands; 5 Department of Clinical Genetics, Leiden University Medical Center, Leiden, The Netherlands; 6 Department of Medical Statistics and Bioinformatics, Leiden University Medical Center, Leiden, The Netherlands; 7 Department of Clinical Genetics, Erasmus MC, University Medical Center, Room Ee-2018, P. O. Box 2040, 3000 CA Rotterdam, The Netherlands

**Keywords:** Lynch syndrome, Prediction models, Colorectal cancer, Hereditary cancer

## Abstract

Until recently, no prediction models for Lynch syndrome (LS) had been validated for *PMS2* mutation carriers. We aimed to evaluate MMRpredict and PREMM5 in a clinical cohort and for *PMS2* mutation carriers specifically. In a retrospective, clinic-based cohort we calculated predictions for LS according to MMRpredict and PREMM5. The area under the operator receiving characteristic curve (AUC) was compared between MMRpredict and PREMM5 for LS patients in general and for different LS genes specifically. Of 734 index patients, 83 (11%) were diagnosed with LS; 23 *MLH1*, 17 *MSH2*, 31 *MSH6* and 12 *PMS2* mutation carriers. Both prediction models performed well for *MLH1* and *MSH2* (AUC 0.80 and 0.83 for PREMM5 and 0.79 for MMRpredict) and fair for *MSH6* mutation carriers (0.69 for PREMM5 and 0.66 for MMRpredict). MMRpredict performed fair for *PMS2* mutation carriers (AUC 0.72), while PREMM5 failed to discriminate *PMS2* mutation carriers from non-mutation carriers (AUC 0.51). The only statistically significant difference between *PMS2* mutation carriers and non-mutation carriers was proximal location of colorectal cancer (77 vs. 28%, p < 0.001). Adding location of colorectal cancer to PREMM5 considerably improved the models performance for *PMS2* mutation carriers (AUC 0.77) and overall (AUC 0.81 vs. 0.72). We validated these results in an external cohort of 376 colorectal cancer patients, including 158 LS patients. MMRpredict and PREMM5 cannot adequately identify *PMS2* mutation carriers. Adding location of colorectal cancer to PREMM5 may improve the performance of this model, which should be validated in larger cohorts.

**Electronic supplementary material:**

The online version of this article (doi:10.1007/s10689-017-0039-1) contains supplementary material, which is available to authorized users.

## Introduction

Lynch syndrome (LS) is a hereditary predisposition to colorectal cancer, endometrial cancer and other extra-colonic cancers at a young age [1, 2]. Morbidity and mortality of LS carriers can be significantly reduced by surveillance programs [3–5]. Therefore identifying LS carriers is of great importance.

LS is caused by a germline mutation in one of the mismatch repair (MMR) genes *MLH1, MSH2, MSH6* or *PMS2*, or in the 3′ end of the *EPCAM* gene and consequent hypermethylation of the *MSH2* promoter region [6–10]. As a result, tumours in LS patients are characterized by microsatellite instability (MSI) and by loss of MMR protein expression in immunohistochemistry (IHC) [11–13]. Analysis of MSI and IHC, combined with *MLH1* promoter methylation analysis to exclude sporadic MMR deficient tumours, are used to identify patients with tumours likely caused by LS [13]. A definite diagnosis of LS is made when a pathogenic germline mutation is found.

The revised Bethesda guidelines were based on a set of diagnostic criteria to select patients eligible for LS screening in tumour tissue. However, due to limited sensitivity, many LS patients will likely be missed by these guidelines [14–17]. Several prediction models, such as MMRpro, MMRpredict and PREMM5 have also been developed to calculate an individual’s probability of carrying a germline MMR mutation [18–20]. These models could aid in the selection of patients at high risk of having LS, for tumour analysis or direct germline mutation analysis. MMRpro is less useful in clinical practice since detailed information of all relatives is needed as input for the model [19]. However, MMRpredict and PREMM_1,2,6_ (a previous version of the newly developed PREMM_5_ model) both performed well in previous evaluations [21–27]. An advantage of PREMM5 is that it can also be used for individuals with extracolonic malignancies and healthy individuals, as opposed to MMRpredict, which can only be used for CRC patients. Until recently, all prediction models for LS were developed with cohorts of patients carrying a *MLH1, MSH2*, or *MSH6* mutation. The recently published PREMM5 model is the only model that included *PMS2* mutation carriers in its development.

In this study we aimed to evaluate MMRpredict and PREMM5 in a clinical cohort and for *PMS2* mutation carriers specifically. Additionally, we aimed to identify clinical features useful for distinguishing *PMS2* mutation carriers from non-mutation carriers.

## Methods

In a retrospective, clinic-based cohort we assessed the performance of MMRpredict and PREMM5 in predicting LS mutations in general and for *MLH1, MSH2, MSH6* and *PMS2* mutations specifically. Additionally, we performed a univariate analysis to identify variables that can distinguish *PMS2* mutation carriers from patients with no MMR mutation.

### Study population

We collected data for all families that were referred for genetic counselling at Erasmus MC, Rotterdam, The Netherlands, and in which colorectal cancer was analysed for MSI and/or IHC between 2000 and 2010. Exclusion criteria were: failed or inconclusive analysis for MSI and IHC, a pathogenic mutation in *APC or MUTYH*, a variant of unknown clinical significance in one of the MMR genes or *APC*, and MSI or IHC suspect for LS while no MMR mutation was detected. To increase the number of LS families, 35 LS families outside our cohort, diagnosed after 2010, were also included in the analysis.

### Analysis of MSI and IHC

MSI analysis was carried out with five markers for MSI as described previously; up to 2007 the Bethesda panel [28] was used and from 2007 onwards our center performs Promega pentaplex MSI analysis [29]. IHC for MLH1, MSH2, MSH6 and PMS2 protein *w*as performed as described previously [13]. Tumours without MSI or only a low degree of MSI and with all MMR proteins present, were considered MMR proficient. Tumours showing a high degree of MSI and/or absence of one or more MMR proteins, were considered MMR deficient. MLH1 hypermethylation analysis was performed to distinguish between sporadic MMR deficient tumours and MMR deficient tumours suspect for LS.

### Germline mutation analysis

Patients with MMR deficient tumours suspect for LS underwent germline mutation analysis of the gene indicated by IHC. Germline mutation analysis of *MLH1, MSH2* and *MSH6* was performed by sequencing and multiplex ligation dependent probe amplification analyses. *PMS2* mutation analysis was performed as described elsewhere [30].

### Family classification

Tumour characteristics, age at diagnosis, results of molecular diagnostics and germline mutation analysis, and a detailed family history were collected from medical records. In every family the patient in whom MSI and/or IHC was analysed, was labelled the index patient. If more than one family member was screened for LS, the youngest CRC patient analysed was considered the index patient. Index patients with MMR proficient tumours or sporadic MMR deficient tumours, were labelled non-mutation carriers. Families identified with a pathogenic MMR mutation were labelled LS families.

### Prediction models

For each index patient the probability of carrying a LS mutation according to MMRpredict and PREMM5 was calculated as previously described [18, 20].

For PREMM5, the equation was slightly different from the published equation, based on personal communications with F. Kastrinos. See Supplemental Material (Appendix 1) for the corrected PREMM5 equation.

### Statistical analysis

Data were analyzed using SPSS statistical software version 21.0. Differences between mutation carriers and non-mutation carriers were compared using the Chi square test or Fishers’ exact test for frequencies, and by using the Mann Whitney *U* test for continuous data. These analysis were also performed to compare *PMS2* mutation carriers with non-mutation carriers. P values < 0.01 were considered statistically significant.

Receiver operating characteristic curves were created for MMRpredict and PREMM5 by plotting the true positive rate (sensitivity) against the false positive rate (1- specificity). Performance of MMRpredict and PREMM5 was evaluated by the area under the receiver operating characteristic curve (AUC). We compared the AUC of PREMM5 and MMRpredict for LS patients in general and for the different MMR genes specifically. Sensitivity and specificity were calculated for cut-offs previously indicated by the developers of the models (5, 10, 20 and 40%). These values were compared with the sensitivity and specificity of the revised Bethesda guidelines.

### Model updating

Location of CRC is included in MMRpredict, but not in the PREMM5 model. To update the PREMM5 model, we used a previously proposed framework to update multinomial logistic regression models [31]. We extended the PREMM5 model using recalibration and extension. The PREMM5 model contains four linear predictors, each contributing weights to the probability of carrying a mutation in *MLH1, MSH2* (or *TACSTD1*), *MSH6* and *PMS2*. The coefficients of the linear predictors were constrained such that the linear predictor only contributed to the calculation of the corresponding mutation. Since the original PREMM5 model was developed on a population with no *MSH6* mutation carriers with two or more CRCs, we developed two adaptations of the PREMM5 model. First we recalibrated the PREMM5 model and re-estimated the coefficient of the predictor ‘Two or more CRCs’ in the linear predictor for *MSH6*. In the second adaptation we also added side of CRC as an additional predictor to the original PREMM5 model. Discriminative ability of the prediction models was quantified using the AUC. Calculations were done using R software (version 3.3.0), with estimation of the coefficients in the updated PREMM5 model using the VGAM package.

### Validation of the extended PREMM5 model

For external validation of the extended PREMM5 model, we used a cohort of 376 CRC patients. Of these patients, 218 were patients with MMR proficient CRC, that where analysed in the Erasmus Medical Center Rotterdam outside the dates of our initial cohort. LS patients (n = 158) in our validation cohort were CRC patients from Leiden University Medical Center in whom an MMR mutation was found and with known location of CRC. For all patients of the validation cohort we calculated the probability of carrying an MMR mutation according to the original PREMM5 model and the extended model. The performance of both models were evaluated by comparing the AUC.

## Results

A total of 734 index patients were included in the study; 346 (47%) were male and mean age at time of diagnosis was 53 years (± 13 years). Overall, 569 (78%) patients fulfilled the revised Bethesda guidelines. Of the 734 index patients, 83 (11%) were diagnosed with a LS mutation; 23 *MLH1*, 17 *MSH2*, 31 *MSH6* and 12 *PMS2* mutation carriers.

### Patient characteristics

Patient characteristics for mutation-positive and mutation-negative patients are shown in Table 1. Significantly more mutation carriers developed multiple CRCs (21 vs. 10%, p = 0.005) and multiple LS-associated cancers in general (13 vs. 4%, p = 0.002) than non-mutation-carriers. CRC patients carrying an MMR mutation had a younger age of onset (49 vs. 53 years, p = 0.002) and more often had proximal CRCs (64 vs. 28%, p < 0.001) than non-mutation carriers. Among women, the frequency of EC was higher for mutation carriers than for non-mutation carriers (41 vs. 3%, p < 0.001). In the mutation positive group, first and second degree relatives developed CRC at a younger age than in the mutation negative group (50 vs. 64 years, p < 0.001 and 47 vs. 62 years, p = 0.008). First degree relatives of mutation carriers had higher rates of EC than relatives of non-mutation carriers (19 vs. 5%, p < 0.001).

**Table 1:** Index characteristics and family history by mutation status (n = 734)

	Mutation negative, % (n)	Mutation positive, % (n)	P value
n	651	83	
Revised Bethesda guidelines	76% (494)	90% (75)	0.003
Index characteristics			
Male gender	47% (305)	49% (41)	0.66
CRC			
Age CRC (median, IQR)	53 years [45–62]	49 years [39–59]	0.002
Proximal CRC	28% (185)	64% (53)	< 0.001
≥ 2 CRCs	10% (66)	21% (17)	0.005
Endometrial cancer	3% (11)	41% (17)	< 0.001
Age EC (median, IQR)	55 years [50–75]	54 years [49–57]	0.18
Multiple LS cancers	4% (27)	13% (11)	0.002
First degree relatives			
CRC	55% (358)	51% (42)	0.45
≥ 2 FDRs with CRC	16% (107)	17% (14)	0.92
Age CRC (median, IQR)	64 years [55–71]	50 years [43–57]	< 0.001
Endometrial cancer	5% (35)	19% (16)	< 0.001
≥ 2 FDRs with EC	0.6% (4)	2% (2)	0.14
Age EC (median, IQR)	55 years [50–64]	50 years [45–57]	0.25
Other LS cancers	22% (142)	19% (16)	0.60
Second degree relatives			
CRC	33% (212)	35% (29)	0.66
≥ 2 SDRs with CRC	12% (81)	12% (10)	0.92
Age CRC (median, IQR)	62 years [50–74]	47 years [38–64]	0.008
Endometrial cancer	3% (22)	7% (6)	0.12
≥ 2 SDRs with EC	0.3% (2)	2% (2)	0.07
Age EC (median, IQR)	70 years [50–76]	49 years [44–51]	0.13
Other LS cancers	16% (104)	18% (15)	0.63

### Discriminative ability of prediction models

**Fig. 1:**
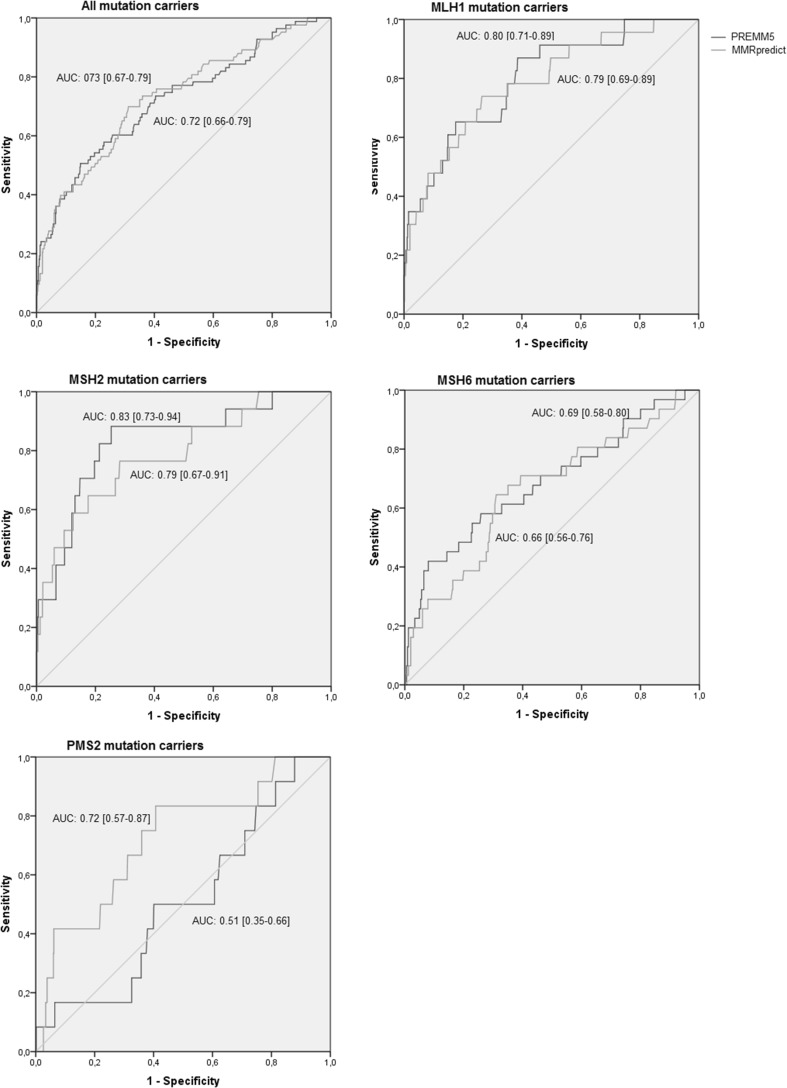
Performance of PREMM5 and MMRpredict in a clinical setting for all mutation carriers and for individual MMR mutations

Overall, PREMM5 predicted higher probabilities of carrying a LS mutation than MMRpredict (median score 0.06 vs. 0.03, Supplemental Table 1). For mutation carriers, risk scores varied from 0.02 to 0.99 for PREMM5 and from 0.002 to 0.99 for MMRpredict. Both prediction models could fairly discriminate between index patients with and without an MMR mutation.(Fig. 1) PREMM5 and MMRpredict had similar overall performance (AUC 0.72 [95% CI 0.66–0.79] vs. 0.73 [95% CI 0.66–0.79]). For *MLH1* and *MSH2* mutation carriers, both prediction models performed well, with AUC of 0.80 [95% CI 0.71–0.89] and 0.83 [95% CI 0.73–0.94] for PREMM5 and AUC of 0.79 [95% CI 0.69–0.89 and 0.67–0.91] for MMRpredict. Both models had a fair discriminative power for *MSH6* mutation carriers (AUC of 0.69 [95% CI 0.58–0.80] for PREMM5 and AUC of 0.66 [95% CI 0.56–0.76] for MMRpredict). MMRpredict still had fair performance for *PMS2* mutation carriers (AUC of 0.72 [95% CI 0.57–0.87]), while PREMM5 failed to discriminate *PMS2* mutation carriers from non-mutation carriers at all with an AUC of 0.51 [95% CI 0.35–0.66].

### Sensitivity and specificity

Using a cut-off of 5% for both prediction models, PREMM5 had a higher sensitivity than MMRpredict (78 vs. 70%). This higher sensitivity came at the expense of a lower specificity (46 vs. 67%). For PREMM5, using a cut-off of 5%, resulted in a sensitivity for *MLH1* and *MSH2* mutations of 88 and 91%, while the sensitivity for *MSH6* mutation carriers was 74% and the sensitivity for *PMS2* mutation carriers was only 50%. For MMRpredict, at a 5% cut-off sensitivity for *MLH1* and *MSH2* mutation carriers were 74 and 77%, while sensitivity for *PMS2* as well as *MSH6* mutation carriers were 65 and 67%. For both models, using a cut-off of ≥ 20% failed to identify over 50% of the mutation carriers.

Sensitivity of the revised Bethesda guidelines decreased from 96% for *MLH1* mutation carriers to 83% for *PMS2* mutation carriers (Supplemental Table 2). Overall, the revised Bethesda guidelines had a sensitivity of 90% with a specificity of 24%. In order to reach the same sensitivity, PREMM5 and MMRpredict had a similar specificity (25%).

### PMS2 mutation carriers versus non-mutation carriers

Mutation carriers differed significantly from non-mutation carriers in many ways (Table 1). In contrast, there were almost no significant differences between *PMS2* mutation carriers and non-mutation carriers. Only one significant difference remained; *PMS2* mutation carriers more often had proximal CRC than patients without an MMR mutation (83 vs. 28%, p < 0.001) (Table 2).

**Table 2:** Index characteristics and family history for PMS2 mutation carriers compared with non-mutation carriers

	Mutation negative, % (n)	PMS2 mutation positive, % (n)	P value
n	651	12	
Revised Bethesda guidelines	76% (494)	83% (10)	0.74
Index characteristics			
Male gender	47% (305)	50% (6)	0.83
CRC			
Age CRC (median, IQR)	53 years [45–62]	46 years [40–61]	0.21
Proximal CRC	28% (185)	83% (10)	< 0.001
≥ 2 CRCs	10% (66)	8% (1)	1.0
Endometrial cancer	3% (11)	0% (0)	1.0
Age EC (median, IQR)	55 years [50–75]		
Multiple LS cancers	4% (27)	0% (0)	1.0
First degree relatives			
CRC	55% (358)	42% (5)	0.36
≥ 2 FDRs with CRC	16% (107)	8% (1)	0.70
Age CRC (median, IQR)	64 years [55–71]	62 years [45–90]	0.68
Endometrial cancer	5% (35)	17% (2)	0.14
≥ 2 FDRs with EC	0.6% (4)	8% (1)	0.88
Age EC (median, IQR)	55 years [50–64]	37 years [–]	0.24
Other LS cancers	22% (142)	8% (1)	0.48
Second degree relatives			
CRC	33% (212)	17% (2)	0.35
≥ 2 SDRs with CRC	12% (81)	8% (1)	1.0
Age CRC (median, IQR)	62 years [50–74]	39 years [39–]	0.12
Endometrial cancer	3% (22)	8% (1)	0.35
≥ 2 SDRs with EC	0.3% (2)	8% (1)	0.05
Age EC (median, IQR)	70 years [50–76]	49 years [–]	0.67
Other LS cancers	16% (104)	17% (2)	1.0

### Improvement of the PREMM5 model

**Fig. 2:**
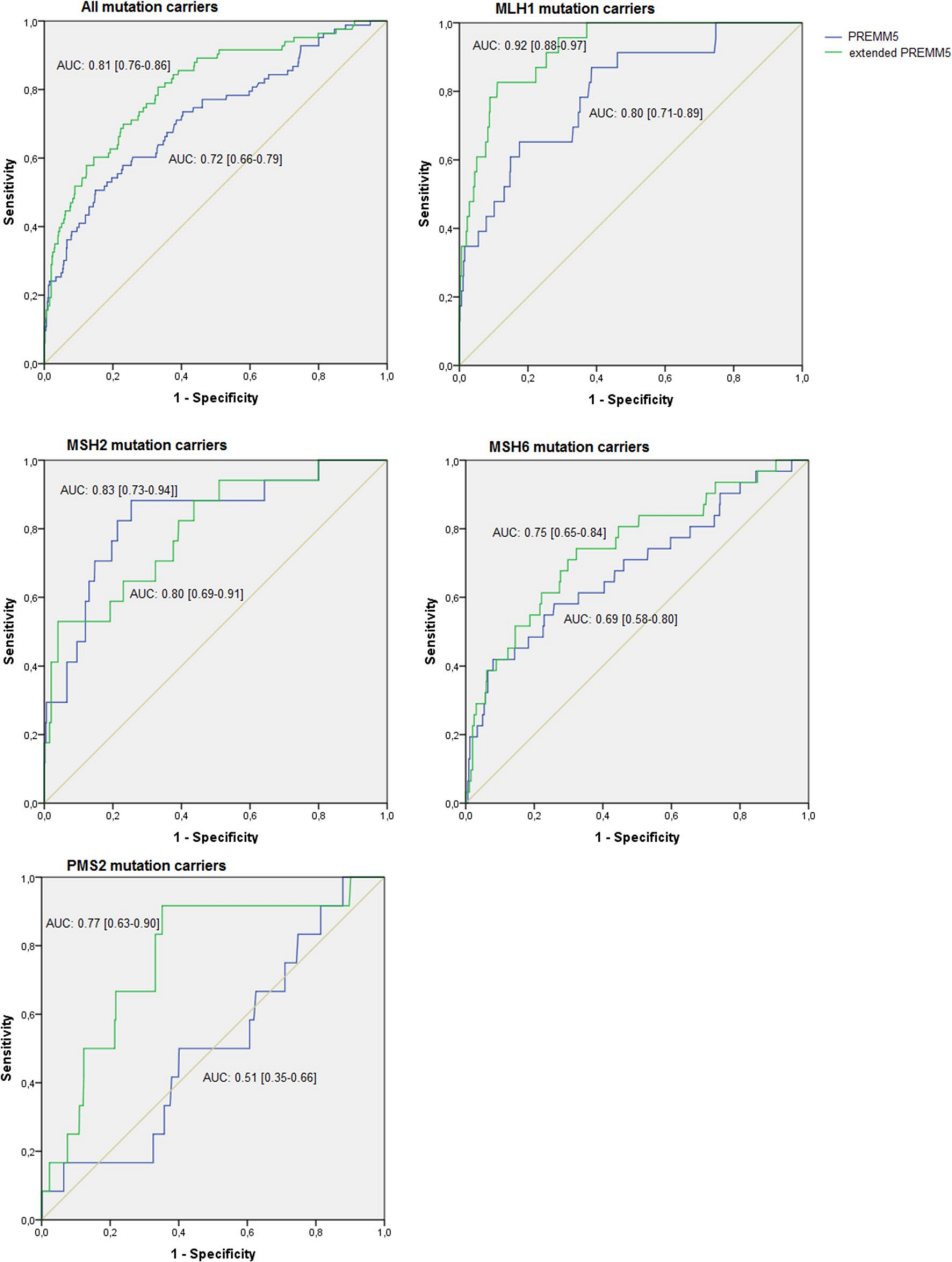
Performance of PREMM5 and the extended PREMM5 model in a clinical setting for all mutation carriers and for individual MMR mutations

Since location of CRC was the only significant difference between *PMS2* mutation carriers and non-mutation carriers, we incorporated this variable in the PREMM5 model, aiming to improve the prediction model. For *PMS2* mutation carriers, the extended PREMM5 model had considerably better predictions than the original PREMM5_5_ model (AUC 0.77 [95% CI 0.63–0.90] vs. 0.51 [95% CI 0.35–0.66]) (Fig. 2). At a 5% cut-off, the new PREMM5 model identified 5/6 *PMS2* mutation carriers that would have been missed by PREMM5 and 3/4 *PMS2* mutation carriers that would have been missed by MMRpredict at the same cut-off.

Adding tumour location also improved the performance of PREMM5 for identifying *MLH1* (AUC 0.92 [95% CI 0.88–0.97] vs. 0.80 [95% CI 0.71–0.89]) and *MSH6* (AUC 0.75 [95% CI 0.65–0.84] vs. 0.69 [95% CI 0.58–0.80]) mutation carriers (Fig. 2). However, performance for *MSH2* mutation carriers slightly decreased (AUC 0.80 [95% CI 0.69–0.91] vs. 0.83 [95% CI 0.73–0.94]). Overall, the adjusted PREMM5 model performed better than the original PREMM5 model (AUC 0.81 [95% CI 0.76–0.86] vs. 0.72 [95% CI 0.66–0.79]) and MMRpredict (AUC 0.81 vs. 0.73 [95% CI 0.66–0.79]). The adjusted prediction model can be found as supplemental material.

At a 5% cut-off, sensitivity of the extended PREMM5 model was higher than the sensitivity of the original PREMM5 model (92 vs. 78%) with similar specificity (45 vs. 46%). Sensitivity and specificity of the extended PREMM5 model at a 5% cut off were both higher than those of the revised Bethesda guidelines (sensitivity 92 vs. 90% and specificity 45 vs. 24%).

### Validation of the extended PREMM5 model

In our validation cohort, 60% of the patients were male and median age was 55 years (IQR 45–63 years). The cohort included 31 *MLH1*, 26 *MSH2*, 28 *MSH6* and 73 *PMS2* mutation carriers. Similar to the results in the initial cohort, the extended PREMM5 model had better predictions than the original PREMM5 model for *PMS2* mutation carriers (AUC 0.90 [95% CI 0.86–0.94] vs. 0.82 [95% CI 0.76–0.87]) and overall (AUC 0.92 [95% CI 0.89–0.95] vs. 0.87 [95% CI 0.84–0.91]). Performance for *MLH1, MSH2* and *MSH6* mutation carriers was also slightly better for the extended PREMM5 model than for the original PREMM5 model (AUC 0.97 [95% CI 0.94–1.00] vs. 0.95 [95% CI 0.91–0.99] for *MLH1*,0.97 [95% CI 0.93–1.00] vs. 0.96 [95% CI 0.92–0.99] for *MSH2* and 0.86 [95% CI 0.97–0.93] vs. 0.85 [95% CI 0.77–0.93] for *MSH6* mutation carriers).

## Discussion

The results of our study indicate that while the models MMRpredict and PREMM5 can adequately predict whether an individual is likely to have Lynch syndrome, they fail to identify *PMS2* mutation carriers. The performance of the PREMM5 model improved considerably by adding the location of CRC to the model. In our clinical cohort of 734 CRC patients as well as in a validation cohort of 376 CRC patients, this extended PREMM5 model not only identified *PMS2* mutation carriers more accurately, its overall performance was also better than the original PREMM5 model and the MMRpredict model.

Our results are in line with those of previous studies, where the PREMM_1,2,6_ model had a slightly better overall performance than MMRpredict [22, 32, 33]. The first PREMM model, PREMM_1,2_ also performed better than MMRpredict in several studies [23, 24], but had similar [25, 26] or less accurate [21] predictions in other studies. A recent meta-analysis also found pooled AUCs to be higher for the PREMM model than for MMRpredict (AUC 0.84 vs. 0.81) [27].

Although PREMM5 had better overall predictions, MMRpredict had a better performance for *PMS2* mutation carriers specifically. An explanation for this could be that the location of CRC is incorporated in the MMRpredict model but not in the PREMM_5_ model. Proximal location of CRC is a known predictor for Lynch syndrome and in our cohort was the only significant difference between *PMS2* mutation carriers and non-mutation carriers. After adding this new variable to the existing PREMM5_5_ model, this new model performed better than MMRpredict for *PMS2* mutation carriers. The extended PREMM5_5_ model also performed better than the original model for *MLH1, MSH2* and *MSH6* mutation carriers and had a better overall performance.

In our validation cohort, all AUCs were much higher than in our original cohort, including those for *PMS2* mutation carriers. Selection of patients for analysis of MSI and IHC may have been less stringent at the Erasmus Medical Center Rotterdam than at the Leiden University Medical Center. Therefore, mutation carriers in our validation cohort, who were all from Leiden University Medical Center, may have had a family history more suspect for Lynch syndrome than family history of the patients in our original cohort. This could explain the higher AUCs in the validation cohort. However, in both cohorts we showed that the extended PREMM5 had better performance.

Prediction models for Lynch syndrome are not yet regularly used in current clinical practice. However, the US Multi-Society Task Force on Colorectal Cancer recommends genetic evaluation if an individual’s risk of carrying an MMR gene mutation is ≥ 5% according to one of the prediction models MMRpro, MMRpredict or PREMM [34]. The American guideline recommends that all CRC patients undergo routine screening for LS by analysis of MSI and IHC [34], while current European guidelines recommend such routine screening in at least all CRC patients up to 70 years of age [35]. A recent study demonstrated that routine screening for LS without an age cut-off is not cost-effective [36]. A strategy using prediction models might lower the cost of screening for LS. In fact, two cost-effectiveness analyses found that strategies including prediction models were more cost-effective than those involving direct tumour testing of all CRC patients, if these prediction models were perfectly implemented [36, 37]. Additionally, prediction models could also be used in cases where no tumour tissue is available or where tumour tissue analysis failed, to assess whether an individual should be analysed for a germline MMR mutation.

The US Multi-Society Task Force on Colorectal Cancer recommends the use of either PREMM, MMRpredict or MMRpro to assess the probability of an individual carrying an MMR mutation [34]. Since we did not include the MMRpro model in our analysis, we do not know how MMRpro would have performed in our cohort. However, MMRpro is less useful in clinical practice since extensive family data is needed as input for the model. Collection of this kind of data is very time consuming and therefore not suitable in clinical practice. PREMM5 and MMRpredict are web-based models that are easily accessible and therefore much easier to use. Also, multiple studies—including the recent meta-analysis—have shown MMRpro to have similar accuracy to PREMM_1,2,6_ [21–27, 32].

Both PREMM5 and MMRpredict were far more accurate for *MLH1* and *MSH2* mutation carriers than for LS patients carrying a mutation in *MSH6* or *PMS2*. This finding is in line with a previous study that showed that carriers of mutations in *MSH6* or *PMS2* had lower risk scores than carriers of a mutation in *MLH1* or *MSH2* [21]. In our study, discrimination between non-mutation carriers and *PMS2* mutation carriers was the least accurate, in line with its more limited penetrance.

Around 15% of all Lynch syndrome cases are estimated to be caused by *PMS2* mutations [38]. In our cohort, 14% (12/83) of the Lynch syndrome patients were *PMS2* mutation carriers. To our knowledge, our study is the first to validate LS prediction models for *PMS2* mutation carriers specifically since the development of the PREMM5 model. At a 5% cut-off, our extended PREMM5 model was able to detect 5/6 *PMS2* mutation carriers who would have been missed by the original PREMM5 model at the same cut-off. Identification of Lynch syndrome carriers is highly important, since this allows not only them, but also their family members carrying the same mutation, to undergo intensive surveillance in order to prevent the development of cancer. Our new model would also identify more Lynch syndrome patients overall than the original PREMM5 model.

The performance of prediction models can differ between high-risk settings and population-based cohorts. Further validation studies should indicate whether our results can be generalized to settings with patients at low to median risk of having Lynch syndrome. Since patients in our study cohort were all referred for genetic counselling, family histories were obtained in detail and in many cases also verified by medical documents. In other settings where patients are at lower risk of having Lynch syndrome, family history is not verified and might be less reliable. Therefore, prediction models should also be validated in population-based cohorts. However, in a meta-analysis, prediction models performed better in population-based cohorts than in clinic-based cohorts [27].

It is not known whether the current prediction models for Lynch syndrome are useful in non-Western populations. In a recent study among Korean patients, PREMM_1,2,6_ was more accurate than MMRpro and MMRpredict, but still only reached an AUC of 0.71 [32]. There was no association between tumour location and mutation status, so our extended PREMM5 model might not improve predictions in populations of non-Western ethnicity. However, germline analysis for *PMS2* was not performed in the Korean study, so there might have been more mutation carriers in their cohort. Another non-Western population has been studied by Khan et al., who analysed the performance of prediction models in 15 African American patients [22]. In these patients, MMRpredict and PREMM_1,2,6_ both had a high AUC of 0.89.

A main strength of our study was the large cohort, which consisted of more than 700 index patient including 83 Lynch syndrome patients. Also, our cohort included patients with *MSH6* and *PMS2* mutations. Since 12 patients were identified as a *PMS2* mutation carrier, we were able to evaluate the prediction models for each MMR mutation specifically, admittedly with considerable uncertainty [39]. Furthermore, we validated the extended PREMM5 model in a separate cohort of 376 patients including 73 *PMS2* mutation carriers.

A limitation of our study was that germline mutation analysis was not done for all index patients. Patients who had microsatellite stable tumours with normal IHC were assumed to be non-mutation carriers. However, some of these patients might still have an MMR mutation. Also, the sample size per gene was still relatively small and it is unclear whether our results from a high-risk population apply to a population-based setting.

In conclusion, we have shown that although MMRpredict and PREMM5 can accurately predict an individual’s risk of carrying a causative MMR mutation, neither model is able to identify patients with *PMS2* mutations. Adding the location of CRC to the PREMM5 model improves the performance of the model for *PMS2* mutation carriers as well as its overall performance. These findings should be validated in large cohorts from population-based settings.

## Electronic supplementary material

Below is the link to the electronic supplementary material.

Supplementary material 1 (DOCX 14 KB)

Supplementary material 2 (DOCX 15 KB)

Supplementary material 3 (DOCX 19 KB)

## References

[1] Lynch HT, de la Chapelle A (2003). Hereditary colorectal cancer. N Engl J Med.

[2] Watson P, Lynch HT (1993). Extracolonic cancer in hereditary nonpolyposis colorectal cancer. Cancer.

[3] de Jong AE, Hendriks YM, Kleibeuker JH (2006). Decrease in mortality in Lynch syndrome families because of surveillance. Gastroenterology.

[4] Jarvinen HJ, Aarnio M, Mustonen H (2000). Controlled 15-year trial on screening for colorectal cancer in families with hereditary nonpolyposis colorectal cancer. Gastroenterology.

[5] Jarvinen HJ, Renkonen-Sinisalo L, Aktan-Collan K, Peltomaki P, Aaltonen LA, Mecklin JP (2009). Ten years after mutation testing for Lynch syndrome: cancer incidence and outcome in mutation-positive and mutation-negative family members. J Clin Oncol.

[6] Akiyama Y, Sato H, Yamada T (1997). Germ-line mutation of the hMSH6/GTBP gene in an atypical hereditary nonpolyposis colorectal cancer kindred. Cancer Res.

[7] Bronner CE, Baker SM, Morrison PT (1994). Mutation in the DNA mismatch repair gene homologue hMLH1 is associated with hereditary non-polyposis colon cancer. Nature.

[8] Fishel R, Lescoe MK, Rao MR (1993). The human mutator gene homolog MSH2 and its association with hereditary nonpolyposis colon cancer. Cell.

[9] Nicolaides NC, Papadopoulos N, Liu B (1994). Mutations of two PMS homologues in hereditary nonpolyposis colon cancer. Nature.

[10] Niessen RC, Hofstra RM, Westers H (2009). Germline hypermethylation of MLH1 and EPCAM deletions are a frequent cause of Lynch syndrome. Genes Chromosomes Cancer.

[11] Aaltonen LA, Salovaara R, Kristo P (1998). Incidence of hereditary nonpolyposis colorectal cancer and the feasibility of molecular screening for the disease. N Engl J Med.

[12] de la Chapelle A (2003). Microsatellite instability. N Engl J Med.

[13] van Lier MG, Wagner A, van Leerdam ME (2010). A review on the molecular diagnostics of Lynch syndrome: a central role for the pathology laboratory. J Cell Mol Med.

[14] Cross DS, Rahm AK, Kauffman TL (2013). Underutilization of Lynch syndrome screening in a multisite study of patients with colorectal cancer. Genet Med.

[15] Julie C, Tresallet C, Brouquet A (2008). Identification in daily practice of patients with Lynch syndrome (hereditary nonpolyposis colorectal cancer): revised Bethesda guidelines-based approach versus molecular screening. Am J Gastroenterol.

[16] Perez-Carbonell L, Ruiz-Ponte C, Guarinos C (2012). Comparison between universal molecular screening for Lynch syndrome and revised Bethesda guidelines in a large population-based cohort of patients with colorectal cancer. Gut.

[17] Van Lier MG, De Wilt JH, Wagemakers JJ (2009). Underutilization of microsatellite instability analysis in colorectal cancer patients at high risk for Lynch syndrome. Scand J Gastroenterol.

[18] Barnetson RA, Tenesa A, Farrington SM (2006). Identification and survival of carriers of mutations in DNA mismatch-repair genes in colon cancer. N Engl J Med.

[19] Chen S, Wang W, Lee S (2006). Prediction of germline mutations and cancer risk in the Lynch syndrome. JAMA.

[20] Kastrinos F, Uno H, Ukaegbu C (2017). Development and Validation of the PREMM5 Model for Comprehensive Risk Assessment of Lynch Syndrome. J Clin Oncol.

[21] Green RC, Parfrey PS, Woods MO, Younghusband HB (2009). Prediction of Lynch syndrome in consecutive patients with colorectal cancer. J Natl Cancer Inst.

[22] Khan O, Blanco A, Conrad P (2011). Performance of Lynch syndrome predictive models in a multi-center US referral population. Am J Gastroenterol.

[23] Monzon JG, Cremin C, Armstrong L (2010). Validation of predictive models for germline mutations in DNA mismatch repair genes in colorectal cancer. Int J Cancer.

[24] Pouchet CJ, Wong N, Chong G (2009). A comparison of models used to predict MLH1, MSH2 and MSH6 mutation carriers. Ann Oncol.

[25] Ramsoekh D, van Leerdam ME, Wagner A, Kuipers EJ, Steyerberg EW (2009). Mutation prediction models in Lynch syndrome: evaluation in a clinical genetic setting. J Med Genet.

[26] Tresallet C, Brouquet A, Julie C (2012). Evaluation of predictive models in daily practice for the identification of patients with Lynch syndrome. Int J Cancer.

[27] Win AK, Macinnis RJ, Dowty JG, Jenkins MA (2013). Criteria and prediction models for mismatch repair gene mutations: a review. J Med Genet.

[28] Boland CR, Thibodeau SN, Hamilton SR (1998). A National Cancer Institute Workshop on Microsatellite Instability for cancer detection and familial predisposition: development of international criteria for the determination of microsatellite instability in colorectal cancer. Cancer Res.

[29] Suraweera N, Duval A, Reperant M (2002). Evaluation of tumor microsatellite instability using five quasimonomorphic mononucleotide repeats and pentaplex PCR. Gastroenterology.

[30] van der Klift HM, Tops CM, Bik EC (2010). Quantification of sequence exchange events between PMS2 and PMS2CL provides a basis for improved mutation scanning of Lynch syndrome patients. Hum Mutat.

[31] Van Hoorde K, Vergouwe Y, Timmerman D, Van Huffel S, Steyerberg EW, Van Calster B (2014). Assessing calibration of multinomial risk prediction models. Stat Med.

[32] Lee SY, Kim DW, Shin YK (2015). Validation of prediction models for mismatch repair gene mutations in Koreans. Cancer Res Treat.

[33] Kastrinos F, Ojha RP, Leenen C et al. (2016) Comparison of prediction models for Lynch syndrome among individuals with colorectal cancer. J Natl Cancer Inst. doi:10.1093/jnci/djv308 10.1093/jnci/djv308 PMC4862416 26582061

[34] Giardiello FM, Allen JI, Axilbund JE (2014). Guidelines on genetic evaluation and management of Lynch syndrome: a consensus statement by the US Multi-Society Task Force on Colorectal Cancer. Gastroenterology.

[35] Vasen HF, Blanco I, Aktan-Collan K (2013). Revised guidelines for the clinical management of Lynch syndrome (HNPCC): recommendations by a group of European experts. Gut.

[36] Barzi A, Sadeghi S, Kattan MW, Meropol NJ (2015) Comparative effectiveness of screening strategies for Lynch syndrome. J Natl Cancer Inst. doi:10.1093/jnci/djv005 10.1093/jnci/djv005 PMC4402362 25794514

[37] Ladabaum U, Wang G, Terdiman J (2011). Strategies to identify the Lynch syndrome among patients with colorectal cancer: a cost-effectiveness analysis. Ann Intern Med.

[38] Palomaki GE, McClain MR, Melillo S, Hampel HL, Thibodeau SN (2009). EGAPP supplementary evidence review: DNA testing strategies aimed at reducing morbidity and mortality from Lynch syndrome. Genet Med.

[39] Vergouwe Y, Steyerberg EW, Eijkemans MJ, Habbema JD (2005). Substantial effective sample sizes were required for external validation studies of predictive logistic regression models. J Clin Epidemiol.

