# Supplementary material for: Evaluation of current prediction models for Lynch syndrome: updating the PREMM5 model to identify PMS2 mutation carriers
Source: Fam Cancer. 2017 Sep 20;17(3):361–70. doi: 10.1007/s10689-017-0039-1 (PMC5999171; doi:10.1007/s10689-017-0039-1)
Supplement: Supplementary file 1 — Supplementary material 1 (DOCX 14 KB) [file 10689_2017_39_MOESM1_ESM.docx]

| **Supplemental Table 1.** Predicted mutation probability with PREMM5 and MMRpredict by mutation status | | | | | | | | |
| --- | --- | --- | --- | --- | --- | --- | --- | --- |
|  |  | Total,  median (IQR) | No mutation,  median (range) | MLH1 mutation,  median (range) | MSH2 Mutation,  median (range) | MSH6 mutation,  median (range) | PMS2 mutation,  median (range) | Any mutation  median (range) |
| n |  | 734 | 651 | 23 | 17 | 31 | 12 | 83 |
|  |  |  |  |  |  |  |  |  |
| PREMM5 |  | 0.06 (0.03-0.12) | 0.04 (0.02-0.09) | 0.16 (0.08-0.44) | 0.17 (0.12-0.71) | 0.11 (0.04-0.28) | 0.06 (0.03-0.08) | 0.14 (0.06-0.32) |
|  |  |  |  |  |  |  |  |  |
| MMRpredict |  | 0.03 (0.01-0.11) | 0.02 (0.01-0.09) | 0.21 (0.05-0.85) | 0.30 (0.05-0.88) | 0.07 (0.02-0.43) | 0.10 (0.04-0.54) | 0.12 (0.03-0.65) |
