# Supplementary material for: Evaluation of current prediction models for Lynch syndrome: updating the PREMM5 model to identify PMS2 mutation carriers
Source: Fam Cancer. 2017 Sep 20;17(3):361–70. doi: 10.1007/s10689-017-0039-1 (PMC5999171; doi:10.1007/s10689-017-0039-1)
Supplement: Supplementary file 2 — Supplementary material 2 (DOCX 15 KB) [file 10689_2017_39_MOESM2_ESM.docx]

| **Supplemental table 2.** Sensitivity and specificity for clinical criteria and prediction models according to different cut-offs | | | | | | | | |
| --- | --- | --- | --- | --- | --- | --- | --- | --- |
| Model | Cut-off | n | All mutation carriers | | MLH1 | MSH2 | MSH6 | PMS2 |
|  |  |  | Sensitivity (%) | Specificity (%) | Sensitivity (%) | Sensitivity (%) | Sensitivity (%) | Sensitivity (%) |
| Revised Bethesda guidelines | - | 569 | 90 | 24 | 96 | 94 | 87 | 83 |
| PREMM_5_ |  |  |  |  |  |  |  |  |
|  | ≥5% | 419 | 78 | 46 | 91 | 88 | 74 | 50 |
|  | ≥10% | 222 | 60 | 74 | 65 | 88 | 58 | 17 |
|  | ≥20% | 89 | 39 | 91 | 44 | 41 | 42 | 17 |
| MMRpredict |  |  |  |  |  |  |  |  |
|  | ≥5% | 276 | 70 | 67 | 74 | 77 | 65 | 67 |
|  | ≥10% | 189 | 53 | 78 | 65 | 65 | 39 | 50 |
|  | ≥20% | 117 | 43 | 88 | 52 | 59 | 29 | 42 |
