# Supplementary material for: Evaluation of current prediction models for Lynch syndrome: updating the PREMM5 model to identify PMS2 mutation carriers
Source: Fam Cancer. 2017 Sep 20;17(3):361–70. doi: 10.1007/s10689-017-0039-1 (PMC5999171; doi:10.1007/s10689-017-0039-1)
Supplement: Supplementary file 3 — Supplementary material 3 (DOCX 19 KB) [file 10689_2017_39_MOESM3_ESM.docx]

**Equation PREMM5 model**

Predicted probability of any mismatch repair gene mutation: p(any)= predicted probability of *MLH1* mutation + predicted probability of *MSH2/EPCAM* mutation + predicted probability of *MSH6* mutation+ predicted probability of *PMS2* mutation.

Predicted probability of a mutation in *MLH1*: p(*MLH1*)

Predicted probability of a mutation in *MSH2* or *EPCAM:* p(*MSH2/EPCAM*)

Predicted probability of a mutation in *MSH6:* p(*MSH6*)

Predicted probability of a mutation in *PMS2:* p(*PMS2*)

Predicted probability of no mutation: p(none) = 1- [p(*MLH1*) + p(*MSH2*) + p(*MSH6*) + p(PMS2)]

p(*MLH1*)= exp (lp(*MLH1*)) / [(1 + exp((lp(*MLH1*)) + exp(lp(*MSH2/EPCAM*)) + exp(lp(*MLH6*)) + exp(lp(PMS2)))]

p(*MSH2/EPCAM*)= exp (lp(*MSH2/EPCAM*)) / [(1 + exp((lp(*MLH1*)) + exp(lp(*MSH2/EPCAM*)) + exp(lp(*MLH6*)) + exp(lp(PMS2)))]

p(*MSH6*)= exp (lp(*MSH6*)) / [(1 + exp((lp(*MLH1*)) + exp(lp(*MSH2/EPCAM*)) + exp(lp(*MLH6*)) + exp(lp(PMS2)))]

p(*PMS2*= exp (lp(*PMS2*)) / [(1 + exp((lp(*MLH1*)) + exp(lp(*MSH2/EPCAM*)) + exp(lp(*MLH6*)) + exp(lp(PMS2)))]

lp(*MLH1*) = -5.402 + (0.901*V0) + (2.586*V1) + (3.171*V2) + (1.620*V3) + (1.275*V4) + (1.578*V5) + (0.804*V6) + (0.391*V7) + (-0.594*(V8/10)) + (0.122*(V9/10)) + (-0.458*(V10/10)).

lp(*MSH2/EPCAM*) = -4.480 + (0.933*V0) + (1.799*V1) + (2.586*V2) + (1.922*V3) + (1.582*V4) + (1.353*V5) + (0.670*V6) + (0.605*V7) + (-0.468*(V8/10)) + (0.004*(V9/10)) + (-0.470*(V10/10)).

lp(*MSH6*) = -4.672 + (0.815*V0) + (1.266*V1) + (-53.181*V2) + (1.755*V3) + (0.536*V4) + (0.549*V5) + (0.916*V6) + (0.315*V7) + (-0.099*(V8/10)) + (0.352*(V9/10)) + (-0.363*(V10/10)).

lp(PMS2) = -4.922 + (0.293*V0) + (0.990*V1) + (-0.353*V2) + (0.739*V3) + (0.394*V4) + (0.003*V5) +

(-0.425*V6) + (-0.105*V7) + (-0.089*(V8/10)) + (0.006*(V9/10)) + (-0.071*(V10/10)).

Equation based on published equation and personal communications with F. Kastrinos.

All variables are equal to the original PREMM5 model: Kastrinos et al, JCO 2017.

**Equation extended PREMM5 model**

Predicted probability of any mismatch repair gene mutation: p(any)= predicted probability of *MLH1* mutation + predicted probability of *MSH2/EPCAM* mutation + predicted probability of *MSH6* mutation+ predicted probability of *PMS2* mutation.

Predicted probability of a mutation in *MLH1*: p(*MLH1*)

Predicted probability of a mutation in *MSH2* or *EPCAM:* p(*MSH2/EPCAM*)

Predicted probability of a mutation in *MSH6:* p(*MSH6*)

Predicted probability of a mutation in *PMS2:* p(*PMS2*)

Predicted probability of no mutation: p(none) = 1- [p(*MLH1*) + p(*MSH2*) + p(*MSH6*) + p(PMS2)]

p(*MLH1*)= exp (lp(*MLH1*)) / [(1 + exp((lp(*MLH1*)) + exp(lp(*MSH2/EPCAM*)) + exp(lp(*MLH6*)) + exp(lp(PMS2)))]

p(*MSH2/EPCAM*)= exp (lp(*MSH2/EPCAM*)) / [(1 + exp((lp(*MLH1*)) + exp(lp(*MSH2/EPCAM*)) + exp(lp(*MLH6*)) + exp(lp(PMS2)))]

p(*MSH6*)= exp (lp(*MSH6*)) / [(1 + exp((lp(*MLH1*)) + exp(lp(*MSH2/EPCAM*)) + exp(lp(*MLH6*)) + exp(lp(PMS2)))]

p(*PMS2*= exp (lp(*PMS2*)) / [(1 + exp((lp(*MLH1*)) + exp(lp(*MSH2/EPCAM*)) + exp(lp(*MLH6*)) + exp(lp(PMS2)))]

lp(*MLH1*) = -7.010 + (0.677*V0) + (1.942*V1) + (2.381*V2) + (1.216*V3) + (0.957*V4) + (1.185*V5) + (0.604*V6) +(0.294*V7) + (-0.446*(V8/10)) + (0.092*(V9/10)) + (-0.344*(V10/10)) + (3.280*V11).

lp(*MSH2/EPCAM*) = -5.269 + (0.868*V0) + (1.674*V1) + (2.407*V2) + (1.789*V3) + (1.472*V4) + (1.259*V5) + (0.624*V6) + (0.563*V7) + (-0.436*(V8/10)) + (0.004*(V9/10)) + (-0.437*(V10/10)) + (0.728*V11).

lp(*MSH6*) = -4.005 + (0.796*V0) + (1.237*V1) + (1.520*V2) + (1.714*V3) + (0.524*V4) + (0.536*V5) + (0.895*V6) + (0.308*V7) + (-0.097*(V8/10)) + (0.344*(V9/10)) + (-0.355*(V10/10)) + (0.868*V11).

lp(PMS2) = -5.511 + (0.040*V0) + (0.134*V1) + (-0.048*V2) + (0.100*V3) + (0.053*V4) + (0.000*V5) +

(-0.058*V6) + (-0.014*V7) + (-0.012*(V8/10)) + (0.001*(V9/10)) + (-0.010*(V10/10)) + (2.540*V11).

V11: Side of colorectal cancer. Enter 0 for left-sided, enter 1 for right-sided.

All other variables are equal to the original PREMM5 model.
